# Integrative Analysis of Nanopore and Illumina Sequencing Reveals Alternative Splicing Complexity in Pig Longissimus Dorsi Muscle

**DOI:** 10.3389/fgene.2022.877646

**Published:** 2022-04-11

**Authors:** Ze Shu, Ligang Wang, Jinbu Wang, Longchao Zhang, Xinhua Hou, Hua Yan, Lixian Wang

**Affiliations:** Key Laboratory of Farm Animal Genetic Resources and Germplasm Innovation of Ministry of Agriculture of China, Institute of Animal Sciences, Chinese Academy of Agricultural Sciences, Beijing, China

**Keywords:** alternative splicing, pig, intramuscular fat, nanopore sequencing, RNA-seq

## Abstract

Alternative splicing (AS) is a key step in the post-transcriptional regulation of gene expression that can affect intramuscular fat (IMF). In this study, longissimus dorsi muscles from 30 pigs in high- and low- IMF groups were used to perform Oxford Nanopore Technologies (ONT) full-length sequencing and Illumina strand-specific RNA-seq. A total of 43,688 full-length transcripts were identified, with 4,322 novel genes and 30,795 novel transcripts. Using AStalavista, a total of 14,728 AS events were detected in the longissimus dorsi muscle. About 17.79% of the genes produced splicing isoforms, in which exon skipping was the most frequent AS event. By analyzing the expression differences of mRNAs and splicing isoforms, we found that differentially expressed mRNAs with splicing isoforms could participate in skeletal muscle development and fatty acid metabolism, which might determine muscle-related traits. *SERBP1*, *MYL1*, *TNNT3*, and *TNNT1* were identified with multiple splicing isoforms, with significant differences in expression. AS events occurring in *IFI6* and *GADD45G* may cause significant differences in gene expression. Other AS events, such as ONT.15153.3, may regulate the function of *ART1* by regulating the expression of different transcripts. Moreover, co-expression and protein-protein interaction (PPI) analysis indicated that several genes (*MRPL27*, *AAR2*, *PYGM*, *PSMD4*, *SCNM1*, and *HNRNPDL*) may be related to intramuscular fat. The splicing isoforms investigated in our research provide a reference for the study of alternative splicing regulation of intramuscular fat deposition.

## Introduction

As pork is the main meat resources for humans, its palatability has always been an important concern. The content of intramuscular fat (IMF) in meat is strongly correlated with the overall meat quality. Increased IMF content not only promotes the sensory attributes (Alfaia et al., 2019), but also affects the flavor and shear force of pork (Zhang et al., 2021). Furthermore, consumers are more likely to accept meat with higher IMF (Malgwi et al., 2022). However, the molecular mechanism and transcriptional regulation process of IMF deposition are still unclear.

Alternative splicing (AS), a key step in the post-transcriptional regulation of gene expression (Lee and Rio, 2015), can directly participate in the pre-transcriptional and post-transcriptional processes and even the processing of RNA and affect the binding between downstream proteins and nucleic acids, proteins, or membranes, and change the localization and enzymatic properties of proteins (Kelemen et al., 2013). AS occurs in different stages of pig development and in different tissues and organs, and affects various economic traits of pig. AS is also involved in the regulation of biological processes and pathways, such as fatty acid degradation (Hao et al., 2022), glyceride metabolism (Li et al., 2012), glucose response (Liu et al., 2010), and fat deposition (Fang et al., 2021).

With the development of the third-generation sequencing (TGS) platform, the nanopore sequencing technology initiated by single-molecule real time (SMRT) technology and Oxford Nanopore Technologies (ONT) (Deamer et al., 2016) have shown great advantages in the identification of AS isoforms (Weirather et al., 2017; Beiki et al., 2019). However, an analysis of ONT-based AS for IMF development in pigs has not yet been conducted.

In this study, we used combined ONT and RNA sequencing analysis to identify potential differential AS factors affecting IMF deposition in pig longissimus dorsi muscle with high and low IMF content. In addition, key candidate genes and AS events affecting IMF deposition were screened by co-expression and PPI analysis. The results can facilitate the study of transcriptional regulatory processes and provide new insights into AS studies in livestock. At the same time, they can be used to provide a new method for improving IMF content in pig breeding.

## Materials and Methods

### Sample Collection and Determination of Intramuscular Fat Content

The animal materials used in this study were all from the F2 generation resource population of large white pig × Min pig at the Changping Experimental Base of the Institute of Animal Sciences, Chinese Academy of Agricultural Sciences. The pig raising and slaughtering environment, tissue collection, and IMF measuring methods were as described in our previous study (Wang et al., 2021). Thirty samples were screened for full-length transcript sequencing based on IMF content: fifteen samples with high IMF content and fifteen samples with low IMF content. The content in the high-IMF group ranged from 4.07% to 12.18%, and that in the low-IMF group ranged from 0.78% to 1.60%. The sample IMF content details are shown in Table 1 and Supplementary Table S13.

**TABLE 1:** Sample IMF content details.

Group	Mean IMF content (%)	SD of IMF content (%)
H	5.48	2.06
L	1.27	0.21
F2	2.85	1.83

### Oxford Nanopore Technologies and Strand-Specific RNA-Seq Library Construction and Sequencing

The ONT library construction was performed according to the standard protocol provided by the ONT platform. Before full-length transcript sequencing, 1 μg of total RNA was prepared for the cDNA library using a cDNA-PCR sequencing kit (SQK-PCS109). The libraries were then created using a sequencing library prep kit. We added the defined PCR adapters directly to both ends of the first-strand cDNA. The cDNA library was established by 20 cycles of PCR amplification with LongAmp Tag. The PCR products were then subjected to ONT adapter ligation using T4 DNA ligase. Agencourt XP beads were used for DNA purification according to the ONT protocol. The final cDNA library was added to the FLO-MIN109 flow cell, and the library was sequenced using the MinION Mk1B sequencer. Strand-specific RNA sequencing data were obtained to validate and quantify the sequencing data of the ONT full-length transcripts. Approximately 2 μg of RNA per sample was used to prepare Illumina sequencing libraries, and 30 prepared libraries were sequenced using the Illumina HiSeq 4,000 platform with a 150 bp pair-end sequencing strategy according to the manufacturer’s instructions.

### Data Analysis of Nanopore Raw Data

For raw data from the Nanopore sequencing fast5 format, Guppy software in the MinKNOW2.2 software package was used to perform base calling and conversion to fastq format. Nanoplot (De Coster et al., 2018) was used to filter the converted data. The clean data were compared with the Silva rRNA database using sortmerna (Kopylova et al., 2012) to delete ribosomal RNA and for subsequent analysis. The sequence containing primers at both ends were determined to be a full-length sequence using pychopper (https://github.com/nanoporetech/pychopper/) software. The obtained full-length sequences were aligned with the pig reference genome (*Sus scrofa* 11.1.96) using minnimap2 software (Li, 2018), and the data were cleaned and clustered using pinfish (https://github.com/nanoporetech/pinfish/) to obtain consistency sequences. cDNA_Cupcake (https://github.com/Magdoll/cDNA_Cupcake/) was used to filter the sequences with identity ≤0.9 and coverage ≤0.85. Alignments with differences in only the 5′ end exons were merged, and 43,668 strips of gff file redundancies were obtained for the sequence information of the remaining transcripts. In addition, gffcompare (https://github.com/gpertea/gffcompare) was used to compare the transcripts obtained from full-length sequencing with the known transcripts of the genome, to discover new genes and transcripts and to supplement the genome annotation.

### Identification of Alternative Splicing Events

To detect all AS events, we generated gff files after de-redundancy and used AStalavista software (Foissac and Sammeth, 2007) to perform AS analysis. Five types of AS events were obtained: exon skipping (ES), intron retention (IR), alternative 5′ splice site (A5SS), alternative 3′ splice site (A3SS), and mutually exclusive exons (MEE). The results of the AStalavista analysis were then transformed to obtain AS event information for all transcripts.

### Validation of the Existence of Alternative Splicing Events by RNA Sanger Sequencing

To verify the accuracy of the identified alternative splicing events, we randomly selected several splicing isoforms for PCR validation. Specific primers upstream and downstream of the alternative splicing were designed using Primer 5. The PCR amplification reaction mixture was 25 μl, including 12.5 μl of Premix Taq (Takara RR902A), 1 μl of template cDNA, 1 μl of each primer, and 9.5 μl of sterile water. PCR products were examined by 1.0% agarose gel electrophoresis and sequenced using the Sanger sequencing method. The primers used in this study are listed in Supplementary Table S12.

### Functional Annotation and Enrichment Analysis of Full-Length Transcripts

To obtain comprehensive functional information annotations for all transcripts, seven databases were searched using BLASTX v.2.2.26 (E-value < 1 × 10^−5^): Gene Ontology (GO, http://www.geneontology.org), Kyoto Encyclopedia of Genes and Genomes (KEGG, http://www.genome.jp/kegg/), Cluster of Orthologous Groups of proteins (COG, http://www.ncbi.nlm.nih.gov/COG/), Clusters of Orthologous Groups for eukaryotic complete genomes (KOG, ftp://ftp.ncbi.nih.gov/pub/COG/KOG/kyva), Non-Redundant Protein Sequences Database (NR, http://www.ncbi.nlm.nih.gov/), Swiss-Prot (http://www.expasy.org/sprot/), and Pfam database (Pfam, http://pfam.xfam.org/) (Finn et al., 2011) to annotate all de-redundant full-length transcripts. The R package clusterProfiler (Yu et al., 2012) was used to perform GO and KEGG pathway enrichment analysis on alternatively spliced genes, and the significant enrichment criterion was a q-value of <0.05.

### Quantitative Gene Expression Analysis, Differentially Expressed Splicing Isoforms, and Differentially Expressed Genes Analysis

The full-length transcripts generated by ONT sequencing were annotated as reference genomes, and the expression levels of all transcripts were further calculated based on the data generated by Illumina sequencing. Hisat2 was used to estimate gene expression levels and to map clean data onto reference sequences. Stringtie with parameter -e was used to limit read alignment processing to estimate and output only assembled transcripts that matched a given reference transcript. We used python script prepDE.py to obtain read count files from the mapping results.

Only the expression data of alternatively spliced transcripts were extracted, and DESeq2 (Love et al., 2014) was used to analyze DESIs and DEGs between the high- and low-IMF groups. The DEseq2 package was used for analysis, the parameter design was set to types and conditions, the type parameter was the sequencing batch, and the conditions parameter was the test grouping. DESIs and DEGs were filtered according to the following criteria: padjust <0.05 and |log_2_ FoldChange| ≥ 1.

### Expression Proportion Analysis and Protein Structure Prediction of Differentially Expressed Splicing Isoforms

In order to explore the regulatory effect of the identified DESIs on gene expression, we analyzed the proportion of all splicing isoforms in their gene expression:

$$\text{Expression proportion of splicing isoforms }\left(\text{count\%}\right)=\frac{\text{splicing isoform reads}}{\text{gene reads}}$$

Protein hydrophobicity (ProtScale, https://web.expasy.org/protscale/) and protein tertiary structure prediction (SWISS-MODEL, https://www.swissmodel.expasy.org/) of amino acid sequences of splicing isoforms were analyzed to explore the potential effect mechanism of AS on gene function.

### Co-Expression and Protein-Protein Interaction Analysis

In this study, to explore the key factors of AS affecting IMF content, we also performed co-expression analysis using weighted gene co-expression network analysis (WGCNA) on all transcripts that were alternatively spliced. Splicing isoforms with a weighted threshold of 0.08 in the screening module were used as hub splicing isoforms. STRING v11.0 (https://string-db.org/) was used to analyze the protein interaction of splicing isoform genes in the module. Cytoscape (version 3.7.2) was used to display the protein interaction map, and the plugin cytoHubba based on degree values was used to identify hub genes in the PPI network.

## Results

### Overview of Oxford Nanopore Technologies and Strand-Specific RNA Sequencing Data

Full-length transcript sequencing was performed on 30 samples, and the clean data generated by each sample sequencing amounted to 5.52 GB on average. The average number of sequences per sample generated by nanopore sequencing was 7,290,257, and the quality values of the samples were all above Q8 (Table 2). A total of 148 GB of raw data was obtained by strand-specific RNA sequencing, and the annotation files obtained by ONT sequencing were aligned and assembled, with an average overall alignment rate of 95.78%. An overview of the next-generation sequencing data is shown in Supplementary Table S1.

**TABLE 2:** Clean reads generated by Nanopore sequencing.

SampleID	ReadNum	BaseNum	N50	MeanLength	MaxLength	MeanQscore
H02	6,752,439	6,175,485,611	962	914	66,795	Q9
H10	5,532,529	5,609,156,050	1,205	1,013	74,639	Q9
H12	6,119,569	6,807,516,576	1,326	1,112	11,721	Q9
H14	6,456,458	6,621,242,447	1,213	1,025	9,744	Q9
H16	5,959,362	5,718,222,829	1,047	959	8,348	Q10
H17	6,603,190	5,758,174,836	925	872	8,386	Q10
H18	7,894,304	8,235,168,413	1,182	1,043	12,564	Q9
H21	7,896,545	7,225,249,807	931	914	15,533	Q10
H22	6,985,943	7,505,345,391	1,252	1,074	15,392	Q9
H24	7,600,605	7,845,590,054	1,129	1,032	12,159	Q9
H25	8,496,048	7,837,286,862	954	922	84,628	Q9
H26	7,469,849	6,585,331,944	904	881	11,670	Q10
H27	5,894,109	5,703,324,956	1,033	967	23,311	Q10
H28	6,376,313	5,618,395,573	908	881	25,245	Q10
H29	5,932,342	5,519,352,014	949	930	13,970	Q10
L04	5,562,596	5,800,700,649	1,215	1,042	13,845	Q9
L06	11,541,467	8,660,383,334	758	750	11,037	Q10
L09	6,491,467	6,103,125,627	1,002	940	10,014	Q9
L14	9,213,725	11,281,078,708	1,426	1,224	15,650	Q8
L16	7,081,487	7,384,134,414	1,227	1,042	13,875	Q9
L19	5,469,901	6,385,532,830	1,378	1,167	11,430	Q9
L21	10,590,964	7,946,674,581	754	750	10,489	Q9
L22	5,890,517	6,526,928,880	1,337	1,108	11,814	Q9
L23	13,079,729	9,621,611,190	744	735	10,721	Q9
L24	5,891,637	5,881,567,295	1,168	998	10,849	Q9
L25	9,107,417	7,198,198,707	781	790	208,750	Q9
L26	6,708,535	5,924,625,514	933	883	9,126	Q9
L27	6,594,848	6,138,091,481	984	930	10,142	Q10
L28	7,043,122	7,191,261,618	1,125	1,021	21,550	Q9
L31	6,470,694	5,937,781,634	936	917	35,868	Q10

ReadNum, number of sequences; BaseNum, the total number of bases; N50, N50 length; MeanLength, average length of reads; MaxLength, longest reads length; MeanQscore, mean quality score.

### Overview of Full-Length Transcripts

**FIGURE 1:**
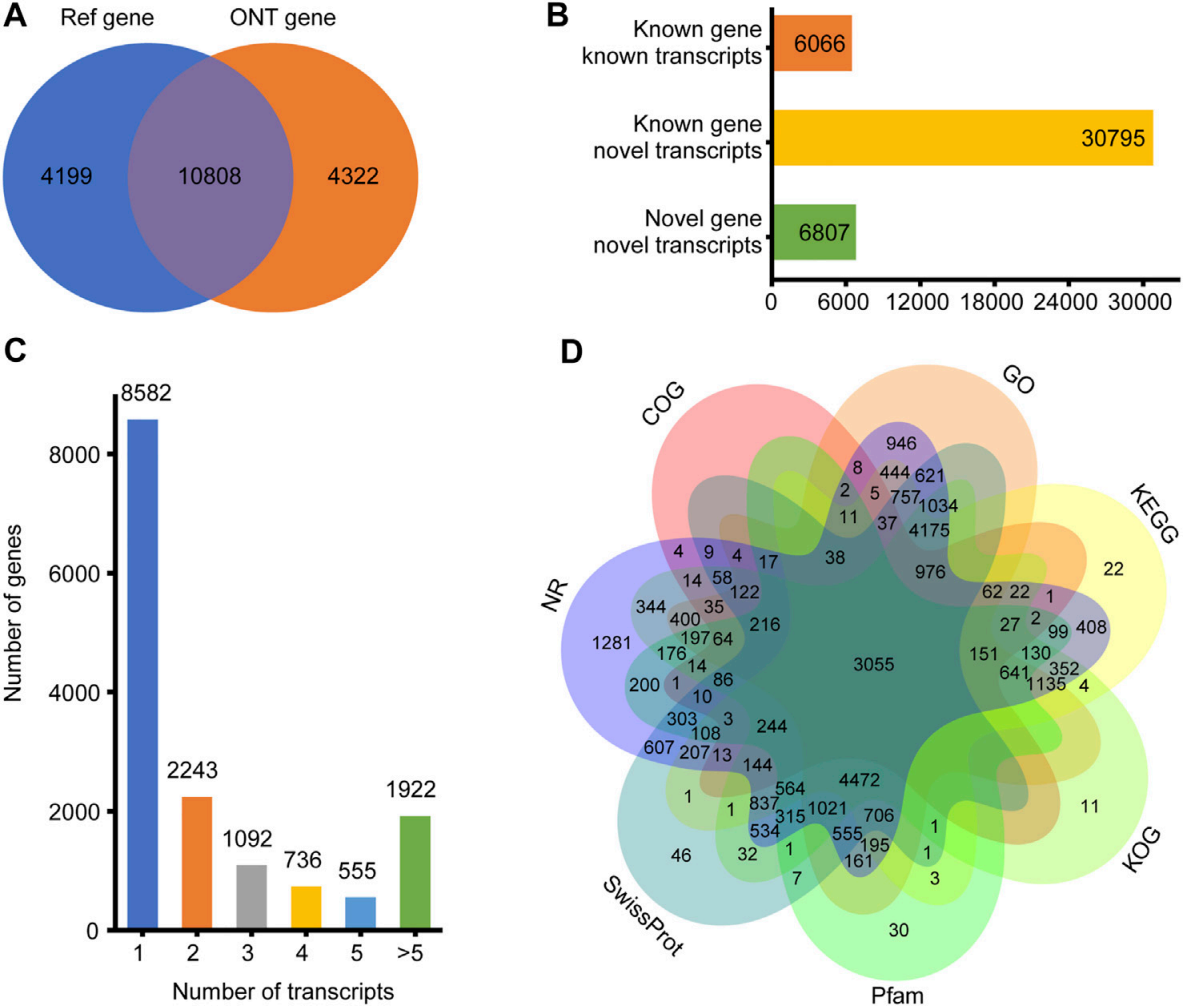
Overview of ONT transcripts. **(A)** Venn diagram of genes identified in ONT data and annotated genomes; **(B)** Distribution diagram of three types of transcripts in ONT data; **(C)** Distribution diagram of gene multi-transcripts; **(D)** Venn diagram of all new transcripts annotation result.

For the 30 individuals, the average number of full-length sequences was 5,081,792 and the average matching proportion of full-length sequences was 79.67%, as shown in Supplementary Table S2. All consensus transcript sequences were aligned to the reference genome by minimap2 software and then subjected to de-redundancy analysis. Finally, 43,688 full-length transcripts were obtained. Mapping of the Sus scrofa genome by gffcompare identified gene loci and transcript isoforms. A total of 15,130 genes were detected; 10,808 (71.43%) known genes were matched and 4,322 (28.57%) genes were newly discovered (Figure 1A). A total of 43,688 transcripts were detected, among which only 6,086 (13.93%) transcripts were known. A total of 30,795 (70.49%) novel transcripts of known genes were identified, and 6,807 novel transcripts of novel genes were identified (15.58%) (Figure 1B). A total of 6,548 (43.27%) genes were identified with two or more transcripts (Figure 1C). Troponin t3, fast skeletal type (TNNT3) had the highest number of transcripts (143 novel and 11 known).

To elucidate their functions, all newly discovered transcripts were annotated to the GO, KEGG, COG, KOG, Pfam, NR, and SwissProt databases. A total of 29,539 transcripts could be annotated in at least one database (Figure 1D).

### Alternative Splicing Identification

**FIGURE 2:**
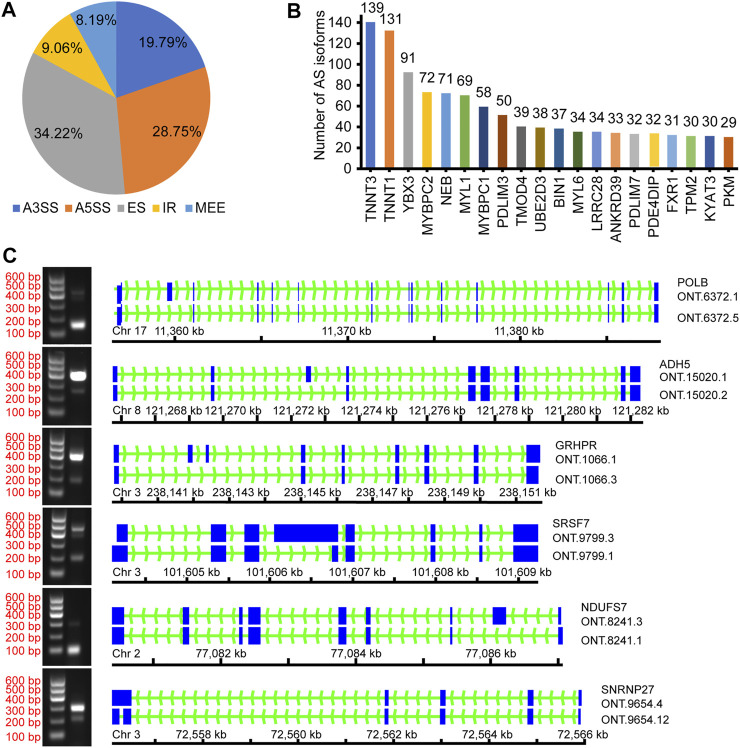
Overview of alternative splicing. **(A)** The proportion of different AS events; **(B)** The number of different gene splicing isoforms; **(C)** Verify different AS events.

AStalavista was used to identify AS of de-redundant transcripts; a total of 10,949 splicing isoforms (25.06%, 43,688 transcripts detected) were generated, and 14,728 AS events were detected (Supplementary Table S4). The largest proportion of AS events was ES (5,040 events, 34.22%), and the smallest proportion was MEE (1,206 events, 8.19%; Figure 2A; Table 3). A total of 2,691 genes (17.79%, 15,130 genes detected by ONT) were alternatively spliced during transcription, and the top five genes with the most splicing isoforms were *TNNT3* (139 splicing isoforms), troponin t1, slow skeletal type (*TNNT1*, 131 splicing isoforms), Y-box binding protein 3 (*YBX3*, 91 splicing isoforms), myosin binding protein C2 (*MYBPC2*, 72 splicing isoforms), and nebulin (*NEB*, 71 splicing isoforms) (Figure 2B).

**TABLE 3:** Number and percentage of AS events.

AS events	Number of AS events	Percentage
Alternative 3′ splice site	2,914	19.79(%)
Alternative 5′ Splice Site	4,234	28.75(%)
Exon skipping	5,040	34.22(%)
Intron retention	1,334	9.06(%)
Mutually exclusive exon	1,206	8.19(%)

### Validation of Selected Alternative Splicing Isoforms

To confirm the accuracy of the identified AS events, we randomly selected several and verified ONT.6273.5, ONT.15020.2, ONT.1066.3, ONT.8241.1, ONT.9799.3, and ONT.9654.4 for validation (Figure 2C). The results support the accuracy of the identified AS events.

### Gene Ontology and Kyoto Encyclopedia of Genes and Genomes Analysis of Genes With Specific Splicing Isoforms

**FIGURE 3:**
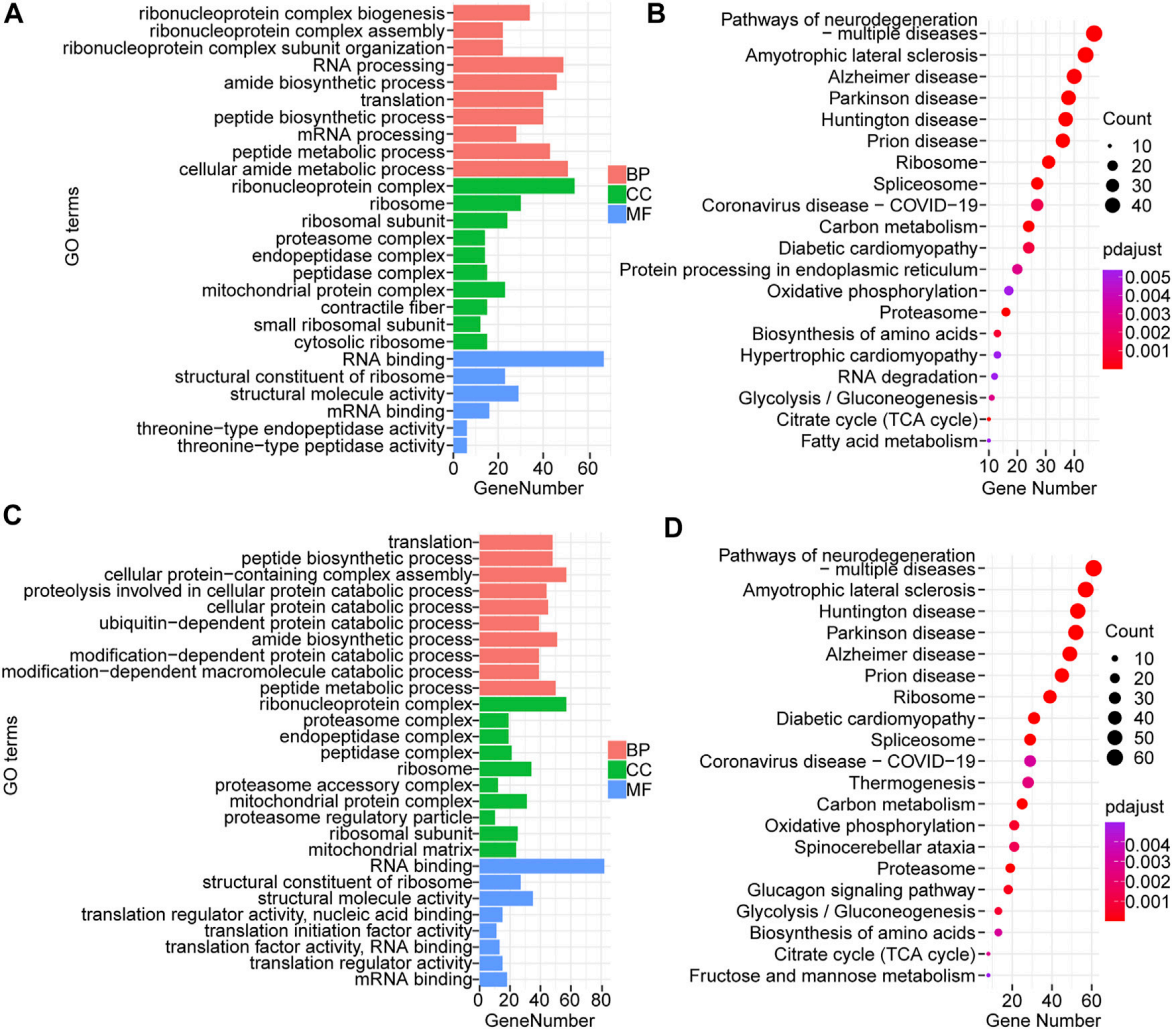
GO and KEGG analysis of splicing isoforms specifically occurring in H and L groups. **(A)** GO results of splicing isoforms that specifically occur in high-IMF group; **(B)** KEGG results of splicing isoforms that occur specifically in high-IMF group; **(C)** GO results of splicing isoforms that specifically occur in low-IMF group; **(D)** KEGG results of splicing isoforms that occur specifically in low-IMF group.

After de-redundancy, we counted the number of de-redundant transcripts for each sample. De-redundant transcripts that were not present in the high- and low-IMF groups but were present in the other group were defined as low- or high-IMF group specific splicing isoforms. In total, 1,605 splicing isoforms were found only in the high-IMF group and 2,235 in the low-IMF group. GO and KEGG analysis was performed on genes of these differentially splicing isoforms using clusterProfiler (Supplementary Table S5). The results indicate that the significant biological process (BP) terms of high-IMF group genes were mainly ribonucleoprotein complex biogenesis, ribonucleoprotein complex assembly, and ribonucleoprotein complex subunit organization; the significant cellular component (CC) terms were ribonucleoprotein complex, proteasome complex, and endopeptidase complex; and the significant molecular function (MF) terms were RNA binding, structural constituent of ribosome, and structural molecule activity (Figure 3A). The enriched KEGG pathways were ribosome, spliceosome, and proteasome (Figure 3B). GO analysis of differentially splicing isoforms in the low-IMF group showed that significant the BP terms were mainly translation, peptide biosynthetic process, and cellular protein-containing complex assembly; significant CC terms were ribonucleoprotein complex, proteasome complex, and endopeptidase complex; and significant MF terms were RNA binding, structural constituent of ribosome, and structural molecule activity (Figure 3C). The enriched KEGG pathways were mainly ribosome, Parkinson disease, and Huntington disease (Figure 3D).

### Differentially Expressed Splicing Isoforms, Differentially Expressed Genes, and Expression Proportion Analysis

**FIGURE 4:**
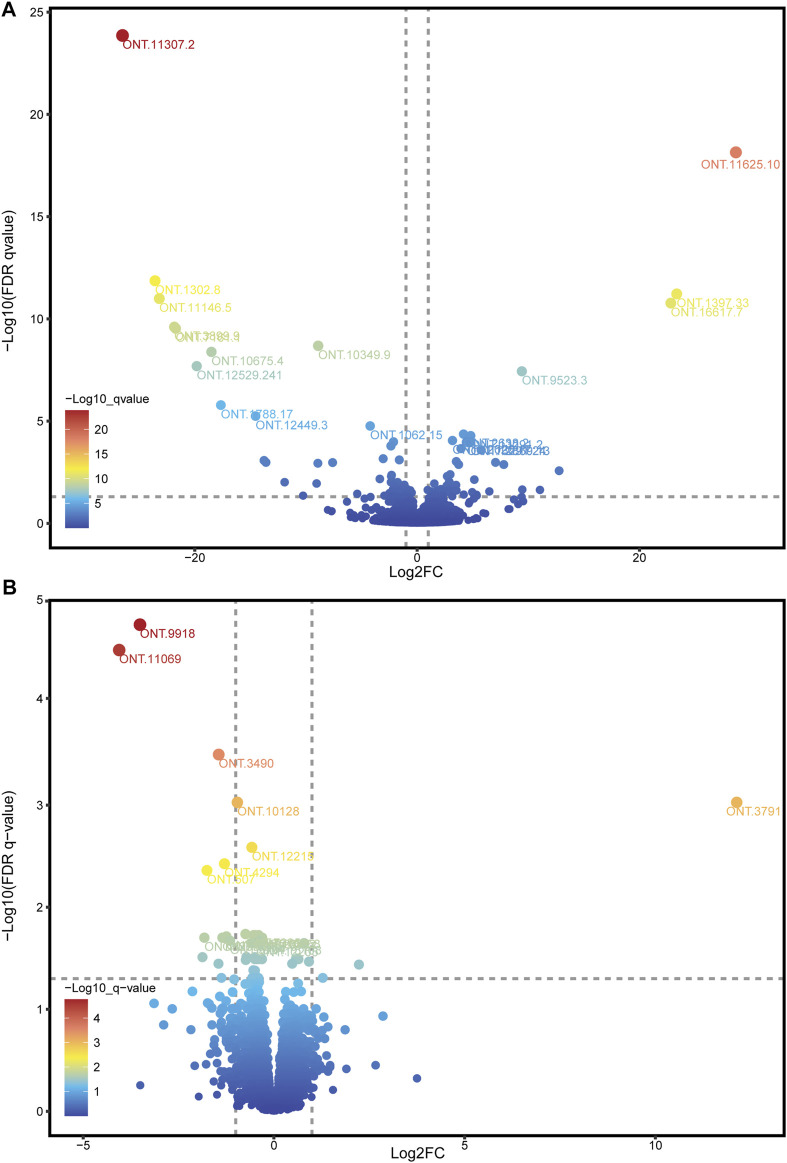
Volcano plot of DESIs and DEGs. **(A)** Volcano plot of DESIs, showing the top 20 splicing isoforms ID with the smallest q-values; **(B)** Volcano plot of DEGs, showing the top 15 genes ID with the smallest q-values.

Because the ONT method has a disadvantage in quantitating expression, we used RNA-seq data to adjust the expression of ONT sequencing data. Combining ONT with RNA-seq data, a total of 10,760 (98.27%) splicing isoform expressions were matched and assembled. Through differential analysis of the expression data of splicing isoforms occurring between high- and low-IMF groups, 98 DESIs (Figure 4A and Supplementary Figure S1), including 49 upregulated and 49 downregulated splicing isoforms, were investigated (Supplementary Table S6). Among all DESIs, SERPINE1 mRNA binding protein 1 (*SERBP1*) had four upregulated splicing isoforms (ONT.13291.2, ONT.13291.18, ONT.13291.21, ONT.13291.24); myosin light chain 1 (*MYL1*) had three upregulated splicing isoforms (ONT.5824.56, ONT.5824.110, ONT.5824.117); and *TNNT3* had three downregulated splicing isoforms (ONT.7235.51, ONT.7235.143, ONT.7235.171). Interestingly, *TNNT1* had two splicing isoforms, with ONT.12529.9 showing an upregulated trend and ONT.12529.241 a downregulated trend.

Through differential analysis of gene expression data between the high- and low-IMF groups, 15 DEGs were identified, including 3 upregulated and 12 downregulated genes (Figure 4B; Supplementary Table S7). Combining the results of DEGs and DESIs, among the downregulated DEGs, ONT.12957 [interferon alpha inducible protein 6 (*IFI6*)] had one transcript (ONT.12957.1) identified in the DESIs results, and ONT.4294 [growth arrest and DNA damage inducible gamma (*GADD45G*)] had two transcripts (ONT 0.4294.3, ONT.4294.9) identified in the DESIs results.

As the ONT sequencing transcripts were long and could cover one gene, we used a criterion to screen the DESIs most likely to be effective, which was the proportion of isoform expression to the expression of all isoforms in the same gene above 10%. As shown in Table 4, we found that ONT.9523.3, ONT.3832.5, ONT.191.5, ONT.15153.3, ONT.11047.3, ONT.16074.8, ONT.10763.6, ONT.7120.12, ONT.7120.13, ONT.14524.8, and ONT.10349.9 had differences in the proportion of expression between high- and low-IMF pigs (*p* < 0.05).

**TABLE 4:** Proportion of splicing isoforms expression in Group H and L.

DESIs	count%_H	count%_L	*p* value
ONT.9523.3	0.1538	0.0263	0.0025
ONT.3832.5	0.2233	0.0777	0.0069
ONT.191.5	0.1054	0.0409	0.0272
ONT.15153.3	0.0373	0.1700	0.0001
ONT.11047.3	0.0392	0.1768	0.0007
ONT.16074.8	0.2267	0.4325	0.0109
ONT.10763.6	0.1634	0.2448	0.0128
ONT.7120.12	0.1288	0.2803	0.0156
ONT.7120.13	0.0505	0.1090	0.0226
ONT.14524.8	0.1720	0.2395	0.0281
ONT.10349.9	0.0005	0.3011	0.0135

### Protein Structural Analysis of Splicing Isoforms

**FIGURE 5:**
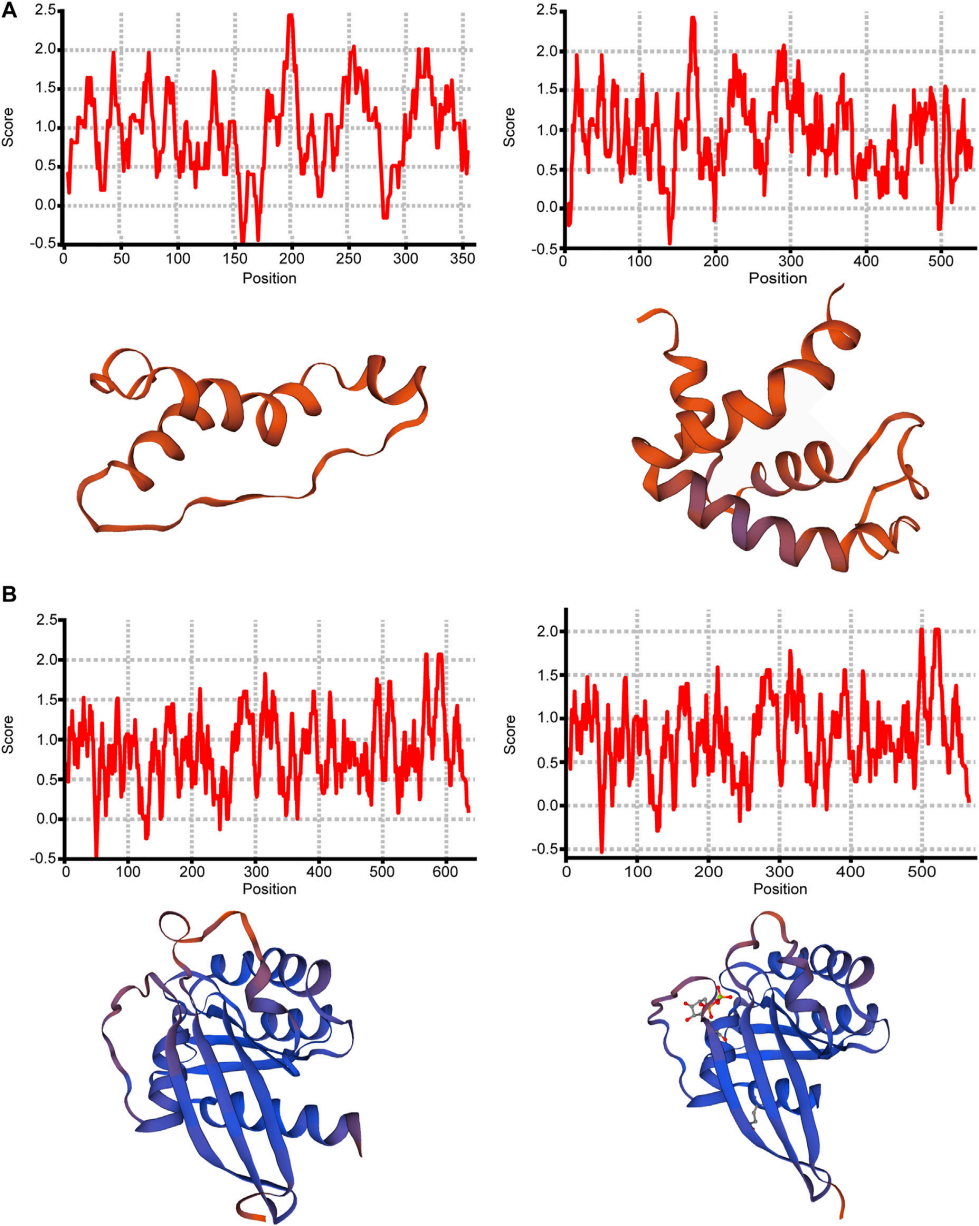
Protein structure analysis of ONT.15153.1, ONT.15153.3, ONT.10349.2 and ONT.10349.9. **(A)** Protein hydrophobicity analysis and protein tertiary structure analysis of ONT.15153.1 and ONT.15153.3; **(B)** Protein hydrophobicity analysis and protein tertiary structure analysis of ONT.10349.2 and ONT.10349.9.

For most of the above 11 isoforms, their expression change could not result in significant expression difference from other transcription expression except ONT.15153.3 and ONT.10349.2, as shown in Supplementary Table S8. From the results, we found that increased expression of ONT.15153.3 will cause reduced expression of ONT.15153.1, and increased expression of ONT.10349.9 will cause reduced expression of ONT.10349.2. We then predicted the protein hydrophobicity and tertiary structure of the genes. We found that the hydrophobicity and tertiary structure of ADP-ribosyltransferase 1 (*ART1*) located in ONT.15153.3 had changed, as shown in Figures 5A,B. Thus, we inferred that the occurrence of AS in *ART1* may have an impact on its protein function, thereby regulating the expression of its gene function.

### Co-Expression Studies of Splicing Isoforms

**FIGURE 6:**
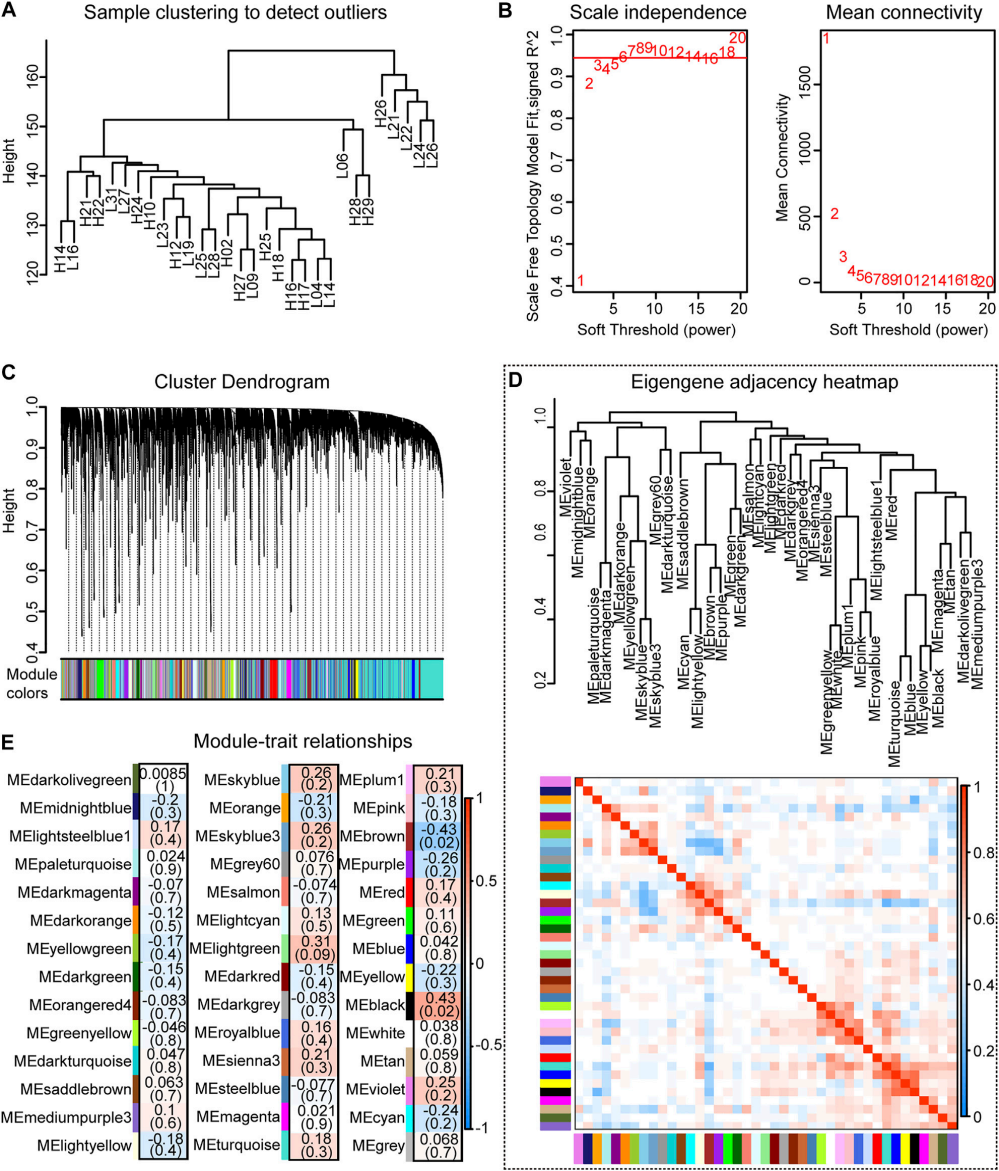
Co-expression analysis results. **(A)** Clustering dendrogram of 30 samples; **(B)** Determination of soft threshold power in co-expression analysis; **(C)** Hierarchical clustering dendrogram of all modules; **(D)** Correlation diagram between modules; **(E)** Relationship between all modules and IMF traits correlation graph.

A co-expression module of the splicing isoforms and IMF deposition was constructed using the WGCNA package tool. By plotting a cluster plot of sample correlations, it could be seen that there were no obvious outlier samples (Figure 6A). We selected the value where the scale-free network graph structure R square reaches 0.95, that is, power = 6 (Figure 6B). By constructing a co-expression network, a total of 42 co-expression modules with different splicing isoforms were identified. Each module was displayed in a different color and contained different numbers of splicing isoforms. Hierarchical clustering and correlation heatmaps were constructed with the 42 modules and correlation heatmaps (Figures 6C,D). Correlation analysis was performed between the co-expression modules and IMF content traits, and correlations and *p*-values between different modules and traits were obtained (Figure 6E).

**FIGURE 7:**
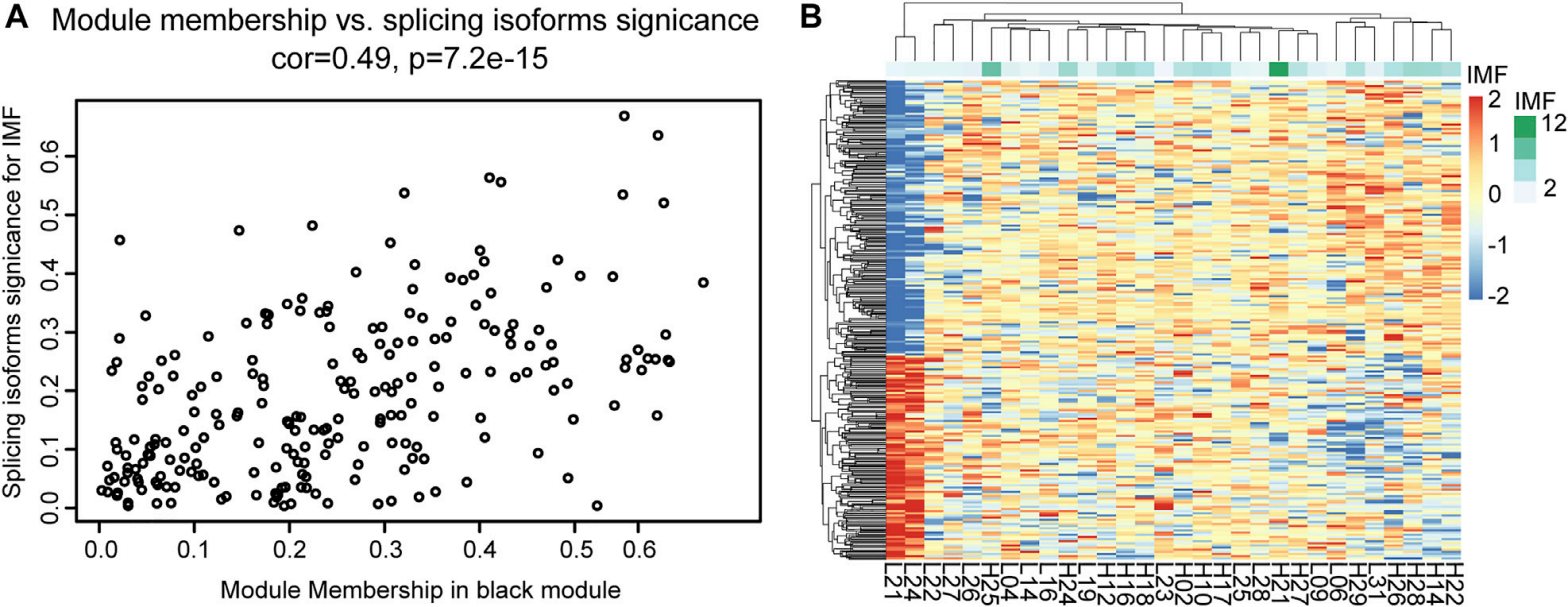
Correlation analysis between MEblack and IMF. **(A)** Dot plot of correlation between splicing isoforms in MEblack and IMF; **(B)** Pheatmap of splicing isoforms in MEblack and 30 samples.

Through co-expression analysis of splicing isoforms, we found a module (MEblack) that was significantly associated with IMF traits. We then plotted the significance of splicing isoform module membership for this module (Figure 7A). At the same time, we also performed correlation analysis on the splicing isoforms in the module and traits (Figure 7B).

**FIGURE 8:**
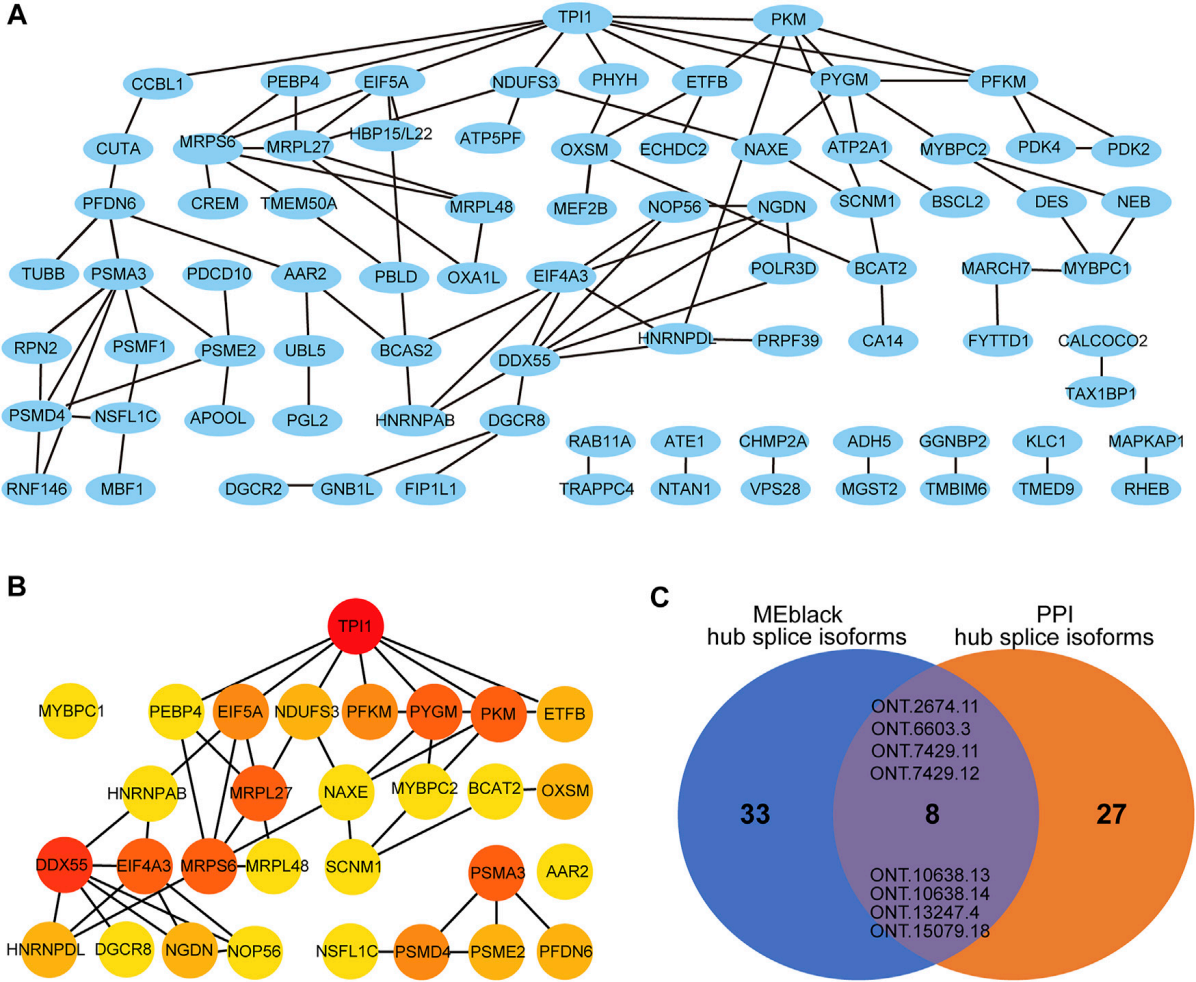
Co-expression and PPI analysis results. **(A)** The gene-PPI network of splicing isoforms in MEblack; **(B)** The PPI network of the Degree top 30 gene; **(C)** The venn diagram of the hub splicing isoforms of MEblack and the hub splicing isoforms of PPI.

Module MEblack aggregated 191 splicing isoforms (Supplementary Table S9), among which 41 hub splicing isoforms were identified by setting a weighted threshold (Supplementary Table S10). Corresponding to 162 named genes, they were imported into the String database to construct a PPI network, and Cytoscape was used to display the PPI results (Figure 8A). Using the cytoHubba plugin, 30 hub genes were identified by the degree method (Figure 8B and Supplementary Table S11). The genes and splicing isoforms were mapped and compared with the hub splicing isoforms screened by the co-expression analysis results, and eight hub splicing isoforms were finally identified (ONT.2674.11, ONT.6603.3, ONT.7429.11, ONT.7429.12, ONT.10638.13, ONT.10638.14, ONT.13247.4, ONT.15079.18) (Figure 8C). The corresponding genes were found to be mitochondrial ribosomal protein L27 (*MRPL27*), AAR2 splicing factor (*AAR2*), glycogen phosphorylase, muscle associated (*PYGM*), proteasome 26S subunit ubiquitin receptor, non-ATPase 4 (*PSMD4*), sodium channel modifier 1 (*SCNM1*), and heterogeneous nuclear ribonucleoprotein D like (*HNRNPDL*).

## Discussion

Full-length transcript sequencing has been shown to be an effective method for studying the post-transcriptional regulation of genes (Cole et al., 2020), and full-length transcripts can be detected by end-to-end sequencing of most transcript sequences, thereby overcoming the errors caused by the complex transcript assembly steps (Ali et al., 2021) required for short-read sequencing. Using ONT sequencing, a total of 43,688 full-length transcripts were identified in this study, 4,322 novel genes and 30,795 novel transcripts. In longissimus dorsi samples, exon skipping and alternative 5′ splice site were the most frequent AS events, accounting for 34.22 and 28.75%, respectively. In a previous study, exon skipping and alternative 5′ splice site were the most common AS events in mammalian pre-mRNA (Sammeth et al., 2008), which is consistent with our study.

The analysis of AS events showed that the five genes with the most splicing isoforms were *TNNT3*, *TNNT1*, *YBX3*, *MYBPC2*, and *NEB*. *TNNT3* and *TNNT1* encode troponin T in fast and slow skeletal muscles, respectively, and play important roles in muscle contraction and regulation of calcium concentration (Wei and Jin, 2016). *YBX3* plays an important role in binding to nucleic acids and transcription factors (Lyabin et al., 2020). *MYBPC2* is involved in the regulation of actin binding, protein binding, muscle structural components, cell adhesion, and myofilament sliding (Chen et al., 2021). *NEB* is a structural component of sarcomeres that extend along filaments and regulate key functions of skeletal muscle (Gineste et al., 2020). In pig longissimus dorsi samples, genes related to skeletal muscle function produced numerous splicing isoforms, and skeletal muscle fibers were also found to be a factor affecting IMF deposition (Veloso et al., 2018), suggesting that AS plays an important role in muscle contraction and activity.

In our study, we also investigated 1,605 and 2,235 splicing isoforms generated only in samples from high-IMF and low-IMF groups, respectively. BP entries enriched in the high-IMF group were significantly different from BP entries enriched in the low-IMF group. BP entries enriched in the high-IMF group were ribonucleoprotein complex assembly, ribonucleoprotein complex subunit organization, and ribonucleoprotein complex biogenesis, while those in the low-IMF group were ubiquitin-dependent protein catabolic process, modification-dependent protein catabolic process, and modification-dependent macromolecule catabolic process. In the KEGG analysis, the significantly enriched pathways in the high- and low-IMF groups were all related to ribosomes, spliceosomes and diseases, glycolysis and gluconeogenesis, and fatty acid metabolism partly caused by abnormal muscle function. The functions of splicing isoforms in the high- and low-IMF groups are mostly related to the regulation of transcriptional processes and muscle function and a series of biological processes of fat synthesis and metabolism. The above results indicate that in the longissimus dorsi of pigs, AS is involved in the regulation of transcription and translation processes, and the spliceosome in the regulation of the occurrence of AS events. AS regulates protein synthesis and catabolism and other processes, which affect the differential functional expression of proteins. At the same time, AS also participates in the regulation of complex processes such as muscle function and fat anabolism.

The expression abundance of all full-length transcripts was obtained by comprehensive analysis of ONT and RNA-seq data. A total of 98 DESIs were identified in high- and low-IMF groups. Four upregulated DESIs were identified for *SERBP1*, which is highly expressed in adipose tissue and regarded as a signal of fat deposition (Duarte et al., 2016). *SERBP1* produces plasminogen activator inhibitor 1 RNA-binding protein (PAIRBP1), an inflammatory adipokine mainly present in adipocytes. Studies have shown that fat deposition can lead to steatosis, triggering the development of fibrosis and steatohepatitis, resulting in upregulation of *PAIRBP1* expression (Ota et al., 2007). In addition, the expression of *SERBP1* can inhibit angiostatin to promote angiogenesis (Nishioka et al., 2011), and the processes of adipogenesis and angiogenesis are synchronized. Our study identified four DESIs of *SERBP1* in the high-IMF group, leading us to speculate that AS may play an important role in IMF deposition by regulating *SERBP1* expression. Moreover, three upregulated DESIs were identified for *MYL1*, which encodes myosin light chain 1. Studies have shown that *MYL3*, the paralogous gene of *MYL1*, plays a negative role in adipogenic differentiation (Zhang et al., 2010). *MYL1* is inversely correlated with muscle tenderness (Gagaoua et al., 2020) and is highly expressed in tougher meats and in pigs with leaner carcasses. *MYL1* is present in fast-twitch fibers, which contain less myoglobin, and ATP is produced by glycolytic metabolism, resulting in more fast-twitch fibers and lower IMF content in tougher meat (Rodrigues et al., 2017). In our study, multiple upregulated DESIs of *MYL1* were identified in samples from the high-IMF group, and we speculated that AS may produce functionally opposite splicing isoforms of *MYL1* to make it function abnormally, and these isoforms may compete for *MYL1* translation substrates and reduce *MYL1* expression in high-IMF samples. This result suggests that AS may enhance gene function or generate functionally opposite splicing isoforms to alter gene expression and function.

Three downregulated DESIs were identified for *TNNT3*, which is involved in encoding fast skeletal muscle fibers, and whose dysfunction contributes to the development of muscular dystrophy symptoms. Castrated male bulls produce more IMF, and *TNNT3* showed a trend of upregulation after castration (Silva et al., 2019). The promotion of IMF deposition by *TNNT3* has been verified in chicken (Liu et al., 2016) and cattle (Bong et al., 2009). Interestingly, a study of the DNA-binding motif of *TNNT3* found that its expression levels were inversely correlated with whole-body fat mass (r_*TNNT3*_ _∼ fat mass_ = −0.49) (Nunez Lopez et al., 2018). This suggests that different splicing isoforms of *TNNT3* play different roles in the process of IMF deposition, which was also confirmed (Schilder et al., 2011). We found in DESIs analysis that *TNNT1* had one upregulated and one downregulated splicing isoform. *TNNT1* is involved in encoding slow skeletal muscle fibers. Studies have found that *TNNT1* is directly associated with obesity traits (Pierzchala et al., 2014), and increased *TNNT1* expression is positively correlated with triglycerides (Nayak et al., 2010). *TNNT1* showed a downward trend in castrated bulls (Silva et al., 2019). The different functions of *TNNT1* may be caused by the opposite functions of different splicing isoforms, which is consistent with the results of our analysis. From the multiple regulatory roles of AS of *MYL1*, *TNNT3*, and *TNNT1*, it can be seen that the splicing patterns of skeletal muscle are diverse and complex, and AS has a critical impact on the development and function of muscle fibers and fat deposition. The expression abundance of different splicing isoforms may promote or reverse the realization of gene function, which greatly enriches proteome diversity.

Combining the results of DEGs and DESIs, it was found that the *IFI6* gene and its splicing isoform ONT.12957.1 were both downregulated in the low-IMF group. The *GADD45G* gene and its splicing isoforms ONT.4294.3 and ONT.4294.9 were also downregulated in the low-IMF group. *IFI6* is a gene that may play an important role in meat quality and carcass indicators and is regarded as a candidate gene for improved pork quality (Kayan et al., 2011). *GADD45G* is an inflammation-related gene associated with oxidative stress and cytokine secretion signaling pathways. The expression of *GADD45G* is upregulated in the liver in high-fat-diet-induced non-alcoholic fatty liver disease (NAFLD), and its expression decreases when the high-fat condition is improved (Wang et al., 2022), which may be related to its effect of on hepatic fibrosis and the occurrence of chemical transformation (Hong et al., 2016). The above results suggest that differential expression of spliced isoforms may cause differences in gene expression.

By analyzing the proportion of expression of each DESI in all transcripts, we explored the important role of splicing isoforms in the realization of gene function. Among the DESIs in the high- and low-IMF groups, we found that the average expression proportion of ONT.9523.3, ONT.3832.5, ONT.15153.3, ONT.11047.3, ONT.16074.8, ONT.10763.6, ONT.7120.12, ONT.7120.13, ONT.14524.8, ONT.10349.9, and ONT.191.5 in samples from the two groups was >10%, and the difference between the two was significant (*p* < 0.05). Then, we compared the expression ratio of splicing isoforms and other transcripts of the gene. The proportion of ONT.15153.1 expression in the low-IMF group was significantly lower than that in the high-IMF group. We speculated that increased expression of ONT.15153.3 would cause decreased expression of ONT.15153.1. At the same time, increased expression of ONT.10349.9 also caused decreased expression of ONT.10349.2. The analysis of its protein hydrophobicity and tertiary structure showed that the occurrence of AS events will lead to different coding information of transcripts, which will change the hydrophobic region of the amino acid sequence, and then affect the conformation of the tertiary structure of the protein. The gene encoding ONT.15153.3 is *ART1*, which catalyzes the ADP-ribosylation of arginine residues in the protein. It is highly expressed in skeletal muscle and is associated with the formation of myotubes and muscle fibers (Leutert et al., 2018). The gene encoding ONT.10349.9 is *RAB2A*, a member of the RAS oncogene family (*RAB2A*) and a membrane-bound protein involved in vesicle fusion and trafficking. *RAB2A* knockdown inhibits glucose-stimulated insulin secretion (Sugawara et al., 2014). According to the above results, the occurrence of AS may change the expression of some transcripts of the same gene, thereby affecting gene function.

Through differential analysis of the expression abundance of alternatively spliced transcripts, it was found that AS plays a variety of regulatory roles in the process of fat deposition. In addition, by constructing a co-expression network, the splicing isoforms of eight centers common to the co-expression and PPI networks were screened, which belonged to six genes: *MRPL27*, *AAR2*, *PYGM*, *PSMD4*, *SCNM1*, and *HNRNPDL*. *MRPL27* encodes a mitochondrial ribosomal protein, and its abnormal function may cause small steric dysfunction and muscle atrophy (Bogatikov et al., 2020). *MRPL27* is also regarded as an NAFLD-related hub gene (Zeng et al., 2021). *AAR2* is a splicing factor that is a component of the U5 snRNP complex and is required for spliceosome assembly and pre-mRNA splicing (Weber et al., 2013). *PYGM* encodes glycogen phosphorylase, which provides energy for muscle contraction, and is associated with glycogen storage in skeletal muscle (Tarnopolsky, 2018). *PSMD4* encodes proteasome 26S subunit ubiquitin receptor, which is a proteasome-related gene that is significantly downregulated in hypercholesterolemia (Loke et al., 2017). *SCNM1* encodes a zinc finger protein and putative splicing factor, and its mis-splicing will lead to serious lesions of sodium channels in mice (Buchner et al., 2003). *HNRNPDL* encodes heterogenetic nuclear ribonucleoprotein d like, which is an important transcription regulator that regulates the AS of hundreds of genes (Li et al., 2019).

## Conclusion

Our study shows that 17.79% of the genes in the longissimus dorsi muscle produced splicing isoforms. Specific splicing isoforms in the high- and low-IMF groups were found to be related to the regulation of the transcription process and muscle fiber function. *MYL1*, *TNNT3*, and *TNNT1* produced different splicing isoforms through AS, which can promote or slow down the process of IMF deposition. The splicing isoforms of *IFI6* and *GADD45G* were differentially expressed, which may cause differences in gene expression, and the expression of the splicing isoforms of *ART1* and *RAB2A* caused changes in the expression of other transcripts. Several genes of hub splicing isoforms, such as *MRPL27*, *AAR2*, *PYGM*, *PSMD4*, *SCNM1*, and *HNRNPDL*, were also investigated with regard to iIMF. These genes may be associated with IMF deposition, but there is currently no direct evidence and further studies are needed to explore their function. Our study preliminarily shows that AS may affect fat deposition in many forms, which also provides further insight into the mechanism of AS regulating IMF content. Our results identified many candidate splice isoforms in IMF that can be used as molecular markers in the breeding and improvement of pig IMF content after validation.

## Data Availability

The datasets presented in this study can be found in online repositories. The names of the repository/repositories and accession number(s) can be found in the article/Supplementary Material.

## Supplementary Material

Supplementary Figure S1 Cluster analysis of all samples with differentially splicing isoforms.

## Glossary

AS: alternative splicing
IMF: intramuscular fat
PPI: protein-protein interaction
ONT: Oxford Nanopore Technologies
PacBio: Pacific Biosciences
TGS: third-generation sequencing
SMRT: single-molecule real time
WGCNA: weighted gene co-expression network analysis
PCR: polymerase chain reaction
ES: exon skipping
IR: intron retention
A5SS: alternative 5′ splice site
A3SS: alternative 3′ splice site
MEE: mutually exclusive exons
GO: Gene Ontology
KEGG: Kyoto Encyclopedia of Genes and Genomes
COG: Cluster of Orthologous Groups of proteins
KOG: Clusters of orthologous groups for eukaryotic complete genomes
NR: NCBI Non-redundant Protein Sequences Database
Pfam: Pfamdatabase
BP: biological process
CC: cellular component
MF: molecular function
DEGs: differentially expressed genes
DESIs: differentially expressed splicing isoforms
TNNT3: troponin t3, fast skeletal type
TNNT1: troponin t1, slow skeletal type
YBX3: Y-box binding protein 3
MYBPC2: myosin binding protein C2
NEB: nebulin
SERBP1: SERPINE1 mRNA binding protein 1
MYL1: myosin light chain 1
IFI6: interferon alpha inducible protein 6
GADD45G: growth arrest and DNA damage inducible gamma
ART1: ADP-ribosyltransferase 1
MRPL27: mitochondrial ribosomal protein L27
AAR2: AAR2 splicing factor
PYGM: glycogen phosphorylase, muscle associated
PSMD4: proteasome 26S subunit ubiquitin receptor, non-ATPase 4
SCNM1: sodium channel modifier 1
HNRNPDL: heterogeneous nuclear ribonucleoprotein D like
PAIRBP1: plasminogen activator inhibitor 1 RNA-binding protein
NAFLD: nonalcoholic fatty liver disease
