# Supplementary figures and images for: Integrative Analysis of Nanopore and Illumina Sequencing Reveals Alternative Splicing Complexity in Pig Longissimus Dorsi Muscle
Source: Front Genet. 2022 Apr 11;13:877646. doi: 10.3389/fgene.2022.877646 (PMC9035893; doi:10.3389/fgene.2022.877646)

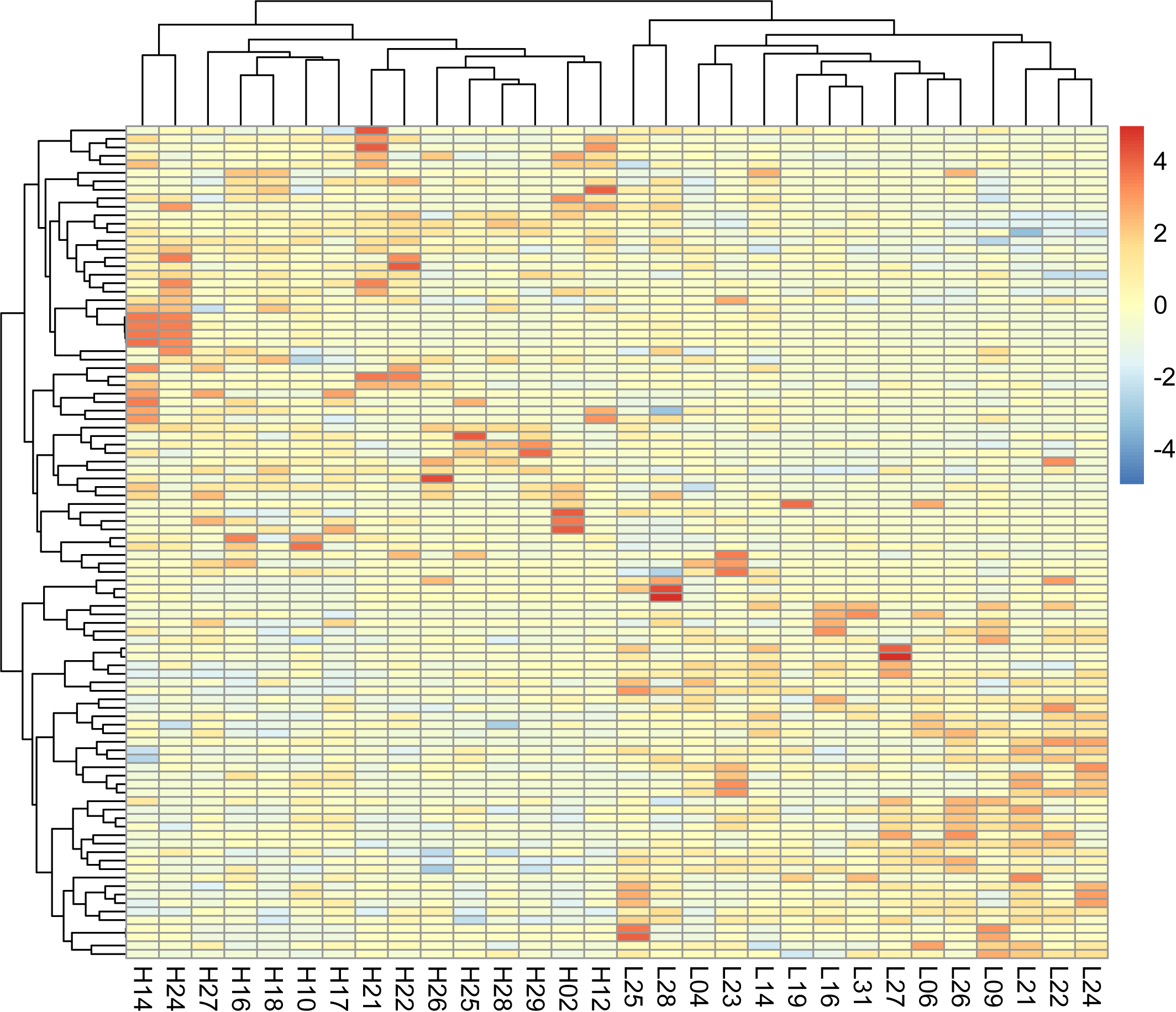

Supplement: Supplementary file 4 [file Image1.TIF]
